# Ion-pair-assisted recovery of nickel(ii) tris(2,2′-bipyridine) complexes from spent catalysts by extraction and precipitation

**DOI:** 10.1039/d6ra08431a

**Published:** 2026-10-22

**Authors:** Hisanori Iwai, Yutaro Takaya, Naoki Yokota, Yuko Takahashi, Chiharu Tokoro

**Affiliations:** a Department of Resources and Environmental Engineering, Faculty of Science and Engineering, Waseda University 169-8555 Tokyo Japan tokoro@waseda.jp; b Department of System Innovation, Faculty of Engineering, The University of Tokyo 113-8656 Tokyo Japan; c Next-Generation Technology Design and Creation, Takahata Precision Co., Ltd 160-0023 Tokyo Japan

## Abstract

Recovery of metals from spent homogeneous catalysts is challenging because strong metal–ligand complexation often prevents conventional inorganic precipitation. In this study, ion-pair-based separation processes were developed for the direct recovery of Ni–2,2′-bipyridine (bpy) complexes from spent catalyst solutions. Perchlorate (ClO_4_^−^) was introduced as a counterion to form the poorly water-soluble ion pair Ni(bpy)_3_(ClO_4_)_2_ (NIP), and ion-pair extraction (IPE) using 1,2-dichloroethane and ion-pair precipitation (IPP) were systematically compared. Alkaline pretreatment was required to adjust the dissolved bpy/Ni ratio to approximately 3, thereby favoring the formation of Ni(bpy)_3_^2+^. Repeated IPE subsequently achieved >99% Ni recovery from both model and actual spent catalyst solutions. In contrast, IPP strongly depended on the initial Ni concentration and solution composition, requiring increasingly large ClO_4_^−^/Ni ratios at lower Ni concentrations. PHREEQC modeling showed that the experimentally determined apparent solubility product governed the onset of NIP precipitation, whereas formation of a dissolved Ni(bpy)_3_(ClO_4_)_2_ ion pair limited the Ni recovery by IPP. ATR-FTIR spectroscopy supported the recovery of the Ni–bpy complex containing ClO_4_^−^ as the counterion. Furthermore, more than 95% of the ClO_4_^−^ counterions were successively exchanged with Cl^−^ using an anion-exchange resin. These results demonstrate that ion-pair separation enables direct recovery of a functional Ni–bpy complex and provides a strategy for closed-loop recycling of homogeneous catalysts.

## Abstract 2

This study demonstrates the direct recovery of intact Ni-bpy complexes from spent homogeneous catalysts *via* ion-pair extraction and precipitation, following by counterion exchange to obtain a more reusable chloride form.

## Graphical abstract

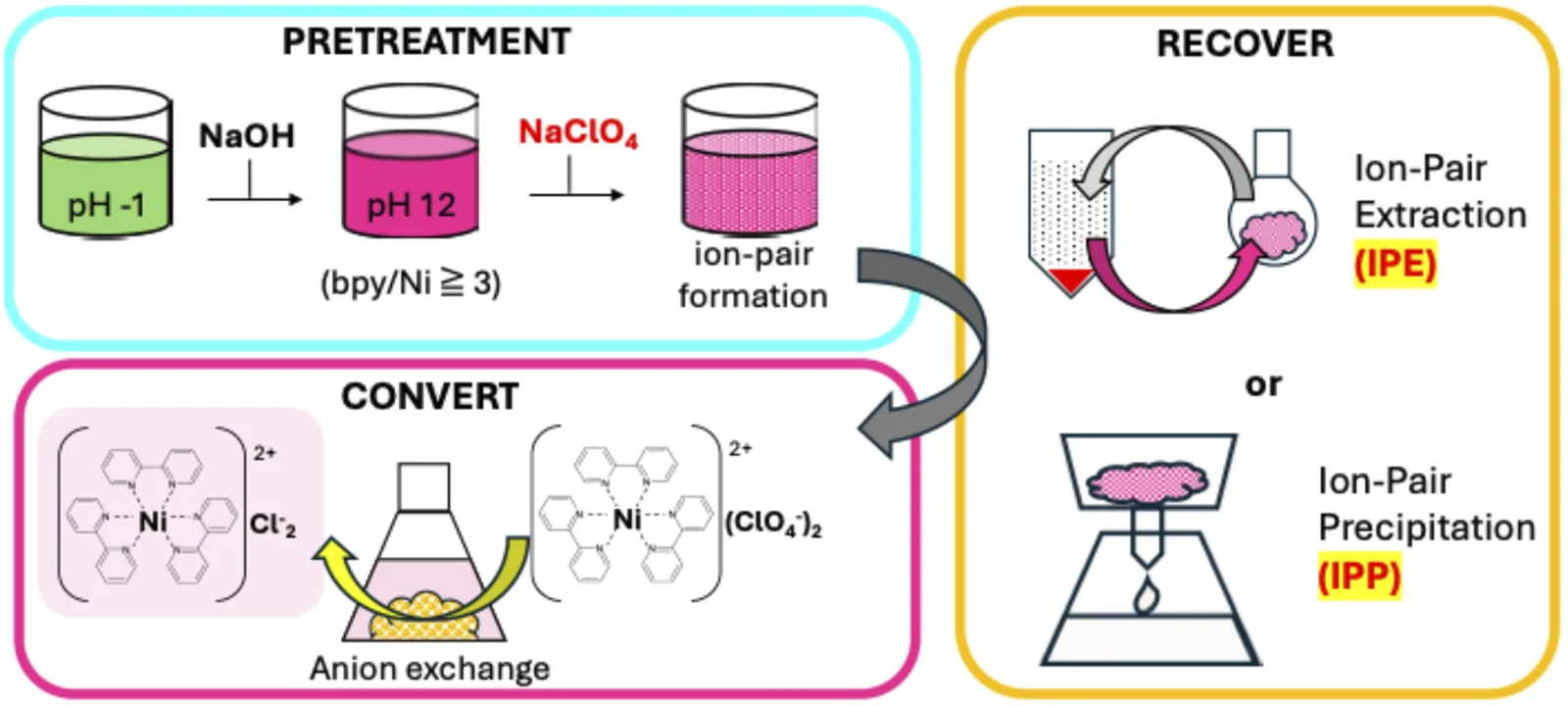

## 1. Introduction

Nickel is an important industrial metal used in alloys, electrochemical devices, and catalytic systems.^1,2^ Growing demand for Ni, particularly in energy-related applications, has increased interest in recovering Ni from secondary resources and establishing more efficient material cycles.^2–4^ Ni is also widely used in homogeneous catalysis. In particular, Ni complexes with 2,2′-bipyridine (bpy) have been employed as catalysts in various chemical transformations, including polymerization and organic synthesis,^5–8^ and have attracted increasing interest in emerging applications such as photocatalytic and electrocatalytic CO_2_ reduction.^9,10^ Although homogeneous catalysts represent a relatively small fraction of the overall Ni demand, spent catalysts constitute a distinct and potentially attractive secondary resource.^2^ Unlike mixed Ni-containing wastes, spent homogeneous catalysts can be collected directly from well-defined laboratory or industrial processes, providing waste streams with known origins and potentially identifiable routes for reuse. These characteristics make spent homogeneous catalysts particularly suitable for localized or closed-loop recycling, in which recovered materials can potentially be returned directly to the processes from which they originated.^11^

Recovery of Ni from spent Ni–bpy catalyst solutions, however, is challenging because of the strong chelation of Ni^2+^ by bpy. Chemical precipitation as metal hydroxides or sulfides is among the conventional approaches for removing and recovering dissolved metals from industrial wastewaters.^2,12,13^ In Ni–bpy systems, the high thermodynamic stability of Ni–bpy complexes substantially decreases the activity of free Ni^2+^, thereby suppressing the formation of conventional inorganic precipitates such as Ni(OH)_2_ and NiS. Our previous study showed that Ni precipitation was strongly suppressed as the bpy/Ni ratio increased and was almost completely inhibited at a bpy/Ni ratio of 3, where Ni(bpy)_3_^2+^ became the predominant dissolved Ni species.^14^ Under other precipitation conditions, interactions among Ni, bpy, and precipitating anions can also result in complex, poorly defined solid phases rather than well-defined inorganic Ni precipitates.^14^ Thus, conventional approaches that require destruction or dissociation of the Ni–bpy complex are not necessarily well suited to the recovery of Ni from spent Ni–bpy catalysts.

As an alternative strategy, direct recovery of the intact metal–ligand complex is particularly attractive for catalyst recycling because it can preserve both the metal and the functional ligand, thereby avoiding ligand decomposition and subsequent resynthesis of the catalyst complex. For spent homogeneous catalysts with well-defined compositions and reuse pathways, preserving the metal–ligand complex thus provides a more direct route to closed-loop recycling than recovering the metal alone.

Ion-pair formation provides a potential means of directly separating charged metal complexes while preserving their coordinated ligands. Association of charged metal complexes with specific counterions substantially alters their solubility and affinity for organic phases, and ion-pair extraction has long been utilized for the analytical separation of metal complexes.^15–17^ Positively charged pyridine-based metal complexes can associate with suitable counter anions to form hydrophobic ion pairs that are extractable into organic solvents such as 1,2-dichloroethane (DCE).^18^ Although ion-pair extraction has been widely employed for analytical separation and trace-metal determination, its application has largely focused on analytical-scale systems. Its extension to the preparative recovery of intact metal–ligand complexes from concentrated process streams remains largely unexplored.

In this study, thus, we developed and evaluated an ion-pair-based separation process for the direct recovery of Ni–bpy complexes from spent catalyst solutions. ClO_4_^−^ was introduced as a counterion to form the poorly water-soluble ion pair Ni(bpy)_3_(ClO_4_)_2_ (denoted as NIP). This study systematically compared two recovery routes, ion-pair extraction (IPE) into DCE and ion-pair precipitation (IPP). The effects of pH, Ni concentration, counterion dosage, and solution composition on Ni recovery were investigated, and the precipitation behavior was quantitatively interpreted using PHREEQC-based chemical equilibrium modeling. Furthermore, the recovered NIP was converted from the ClO_4_^−^ form to a water-soluble Cl^−^ form using an anion-exchange resin while retaining the characteristic spectral features of coordinated bpy.

## 2. Materials and methods

### 2.1. Chemicals

2,2′-Bipyridine (bpy, 98% purity) was obtained from TCI (Tokyo, Japan). Nickel chloride hexahydrate (NiCl_2_·6H_2_O), methanol (MeOH), sodium perchlorate (NaClO_4_), DCE, *N*,*N*-dimethylacetamide (DMAc), and all other chemicals were of analytical reagent grade and obtained from FUJIFILM Wako Pure Chemical Corporation (Osaka, Japan). All chemicals were used without further purification. Reference Ni(bpy)_3_Cl_2_ complexes was synthesized with reference to previous reports.^14,19^ NiCl_2_ and bpy were dissolved in MeOH at bpy/Ni molar ratios of 1 and 3, respectively. MeOH was removed by gentle heating at 60 °C, and the resulting solids were further dried under vacuum for several days to remove residual MeOH.

An actual spent Ni–bpy catalyst solution (SpC-A) generated after polymerization was obtained from TAKAHATA PRECISION Co., Ltd (Tokyo, Japan). Model spent catalyst solutions (SpC-1 and SpC-3) were prepared by dissolving NiCl_2_ and bpy in a 50% MeOH–3 M HCl medium. The initial Ni concentration was 40 mM, with bpy/Ni molar ratios of 1 and 3 for SpC-1 and SpC-3, respectively.^14^ The chemical compositions of these samples are summarized in Table 1.

**Table 1:** Chemical compositions of the actual and synthesized spent catalysts^14^

Components^a^ (mM)	SpC-A	Synthesized spent catalyst	
		SpC-1	SpC-3
NiCl_2_	40	40	40
2,2′-Bipyridine	40	40	120
1,5-Cyclooctadiene	90	—	—
*N*,*N*-Dimethylacetamide	900	—	—

a 50% MeOH 3 M HCl mixture was used as the media. — Not added.

### 2.2. Ion-pair extraction (IPE)

SpC-3 and SpC-A solutions (initial Ni concentration, 40 mM) were adjusted to pH 12 by adding approximately 10 M NaOH solution and stirred for 1 day. To investigate the effect of pH on extraction, model solutions containing 40 mM Ni at bpy/Ni molar ratios of 1 and 3 were adjusted to pH 1, 4, 8, or 12. Precipitated NaCl and Ni(OH)_2_ were removed by centrifugation (8000 rpm, 10 min), followed by filtration through a 0.45 µm PTFE membrane filter.

A 10 mL aliquot of the filtrate was mixed with a predetermined volume of 5 M NaClO_4_ solution and subsequently extracted with 1 mL of DCE. The DCE phase was separated, and the extraction was repeated using fresh DCE. After each extraction, a 100 µL aliquot of the aqueous phase was collected, diluted 100-fold with 1 wt% HNO_3_, and analyzed for Ni by ICP-OES (iCAP 6000; Thermo Fisher Scientific, MA, USA). The cumulative Ni recovery efficiency by IPE (*R*_IPE_, %) was calculated using the following equation:
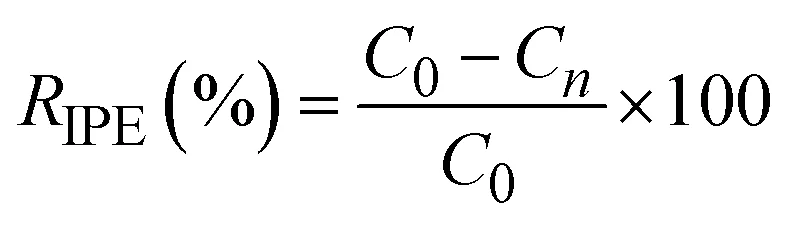
where *C*_0_ and *C*_*n*_ are the Ni concentrations before and after the *n*th extraction, respectively. All Ni concentrations used for the calculation were corrected for changes in the aqueous-phase volume caused by the addition of the NaClO_4_ solution.

DCE was removed from the collected organic phases using a rotary evaporator, and the recovered NIP was characterized by attenuated total reflectance Fourier-transform infrared spectroscopy (ATR-FTIR; 4500a, Agilent Technologies, CA, USA).

### 2.3. Ion-pair precipitation (IPP)

SpC-3 and SpC-A were adjusted to pH 12 and pretreated as described in Section 2.2. A predetermined volume of 5 M NaClO_4_ solution was added to the resulting filtrates. After mixing, the suspensions were allowed to stand at room temperature for 1 day to induce NIP precipitation. The Ni recovery efficiency by IPP (*R*_IPP_, %) was calculated using the following equation:
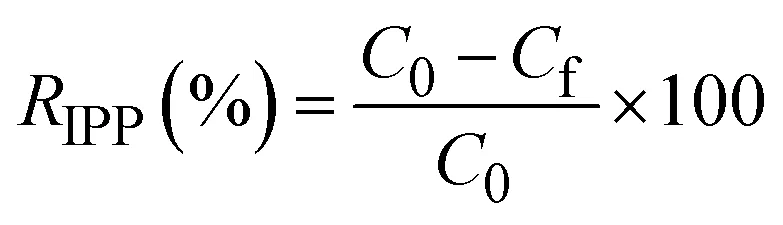
where *C*_f_ is the final Ni concentration after IPP. To investigate the effect of the initial Ni concentration, the pretreated SpC-3 filtrate was diluted to predetermined Ni concentrations using a NaCl-saturated 50% MeOH–0.01 M NaOH medium. After incubation for 1 day, the suspensions were filtered through a 0.45 µm PTFE membrane filter to separate the precipitates. The filtrates were diluted 100-fold with 1 wt% HNO_3_, and the Ni concentrations were determined by ICP-OES. The recovered precipitates were freeze-dried and characterized by ATR-FTIR spectroscopy.

The overall experimental procedures for IPE and IPP are schematically summarized in Fig. 1.

**Fig. 1:**
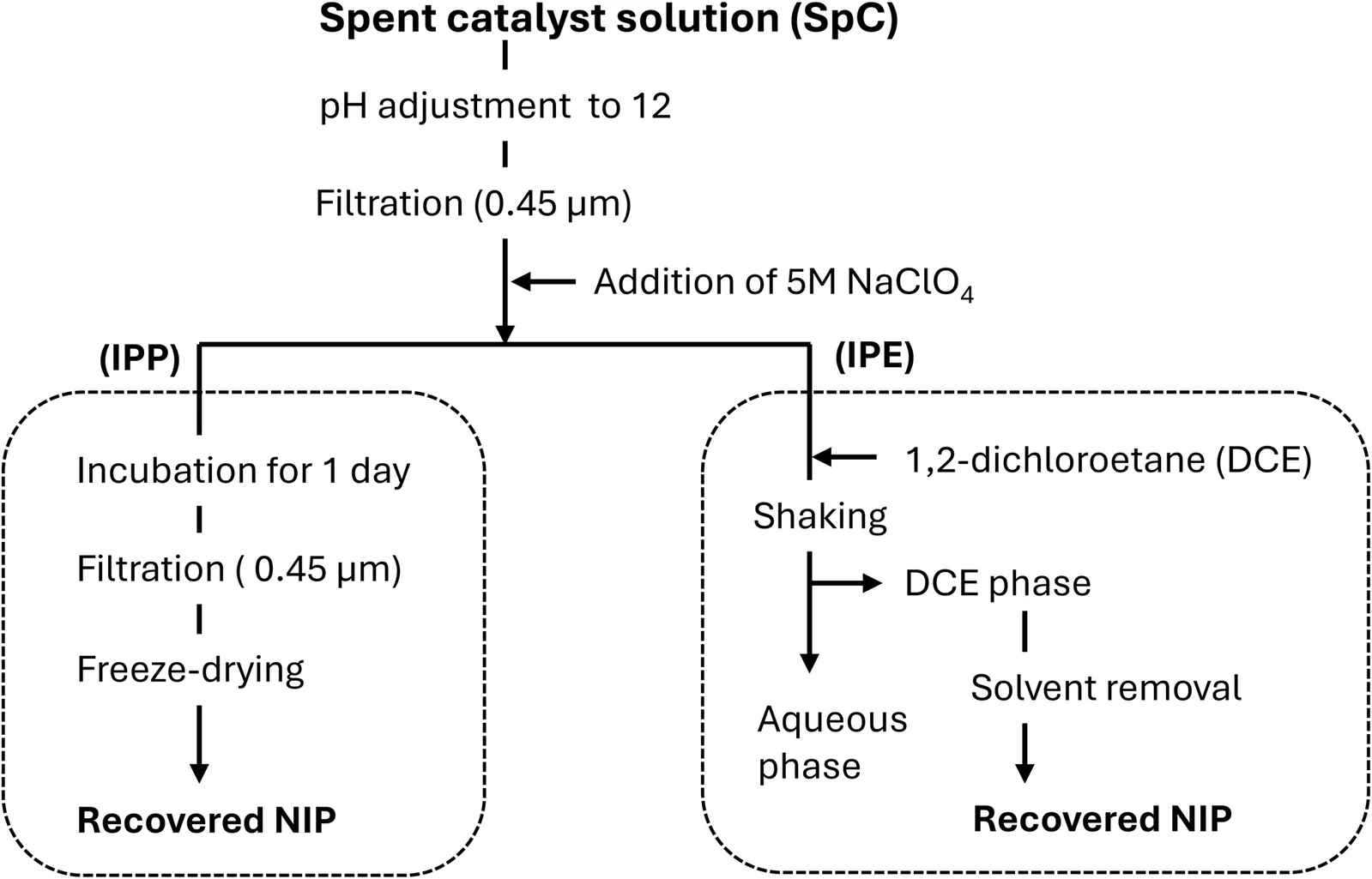
Schematic flow diagram of Ni–bpy complex recovery by ion-pair precipitation (IPP) and ion-pair extraction (IPE). For IPE, sequential extraction with fresh DCE was repeated as required to achieve high Ni recovery. The recovered DCE phases were combined, and DCE was removed by rotary evaporation to obtain NIP.

### 2.4. Chemical modeling

The precipitation behavior of NIP under different conditions, including the initial Ni concentration, solvent composition, and ClO_4_^−^ concentration, was simulated using PHREEQC ver. 3 with the llnl database.^20^ The formation of Ni–bpy complexes was incorporated into the database using the following equilibrium reactions:Ni^2+^ + bpy ⇄ Ni(bpy)_1_^2+^, log *β*_1_ = 6.92,Ni^2+^ + 2bpy ⇄ Ni(bpy)_2_^2+^, log *β*_2_ = 13.69,Ni^2+^ + 3bpy ⇄ Ni(bpy)_3_^2+^, log *β*_3_ = 19.58,where the cumulative stability constants (log *β*_1,2,3_) were defined based on the values reported by Cabani and Landucci.^21^ NIP precipitation was represented by the following dissolution equilibrium:Ni(bpy)_3_(ClO_4_)_2_ ⇄ Ni(bpy)_3_^2+^ + 2ClO_4_^−^.

An apparent solubility product (*K*_sp,app_) was defined as*K*_sp,app_ = [Ni(bpy)_3_^2+^] × [ClO_4_^−^]^2^.

Because NIP solubility varied with the solvent composition, *K*_sp,app_ was experimentally determined for each medium and is reported throughout this study in logarithmic form as log *K*_sp,app_. An excess amount of NIP precipitate prepared from SpC-3 by the IPP method was added to media with different pH values, MeOH contents, NaCl concentrations, and DMAc concentrations and equilibrated for 1 day at room temperature. The dissolved Ni concentration was determined by ICP-OES. Assuming stoichiometric dissolution of NIP, the dissolved ClO_4_^−^ concentration was calculated as twice the dissolved Ni concentration, and log *K*_sp,app_ was determined from these concentrations. The experimentally determined log *K*_sp,app_ values were subsequently used in the PHREEQC calculations.

### 2.5. Exchange of ClO_4_^−^ with C^−^ using an anion-exchange resin

To improve the water solubility and reusability of the recovered NIP, counterion exchange from ClO_4_^−^ to Cl^−^ was performed using Amberlite IRA402BL anion-exchange resin in the Cl^−^ form. According to the supplier, the resin has a particle size of 0.50–0.65 mm and an ion-exchange capacity of ≥1.25 eq L^−1^-resin. The wet resin mass per unit volume was experimentally determined to be 0.7935 g mL^−1^.

For the counterion-exchange experiments, 0.5 g of NIP and 0.25–5 g of wet resin were added to 50 mL of 90% MeOH and stirred for 1 h. After treatment, the resin was removed by filtration through a 0.45 µm membrane filter and rinsed with ultrapure water. MeOH was removed from the filtrate by heating at 60 °C or using a rotary evaporator, and the remaining aqueous solution was adjusted to a final volume of 50 mL with ultrapure water.

To evaluate the Cl^−^ exchange efficiency, a 10 mL aliquot of the resulting solution was subjected to five sequential extractions with 1 mL of DCE. Because the ClO_4_^−^ form of the Ni–bpy ion pair was extracted into DCE whereas the Cl^−^ form remained in the aqueous phase, the apparent Cl^−^ exchange efficiency was determined from the aqueous Ni concentrations before and after DCE extraction using the following equation:
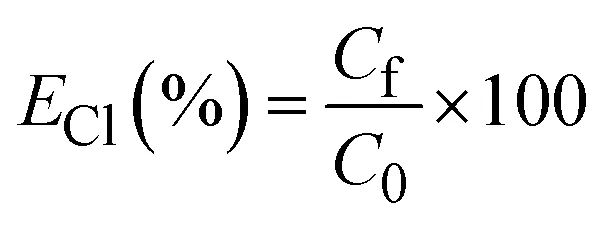
where *C*_0_ and *C*_f_ are the aqueous Ni concentrations before and after five sequential DCE extractions, respectively. Ni concentrations were determined by ICP-OES. The remaining aqueous solution was freeze-dried, and residual ClO_4_^−^ in the recovered solid was evaluated by ATR-FTIR spectroscopy.

## 3. Results and discussion

### 3.1. Effect of pH on Ni recovery by IPE

Fig. 2 shows the effect of pH on Ni recovery by IPE from SpC-1 and SpC-3. For SpC-1, Ni recovery was low under acidic conditions. At pH 0–4, approximately 10% of Ni was recovered in the first extraction, and the cumulative recovery remained below 20% after six extractions. Recovery increased slightly at pH 8, reaching approximately 36% after six extractions. In contrast, at pH 12, approximately 50% of Ni was recovered in the first extraction, >80% after two extractions, and >99% after four extractions. The strong pH dependence of SpC-1 can be attributed to changes in Ni–bpy speciation during alkaline pretreatment. Because SpC-1 had an initial bpy/Ni molar ratio of 1, Ni species with low bpy coordination numbers were expected to predominate before alkaline treatment and exhibit limited extractability into DCE. With increasing pH, uncomplexed or weakly complexed Ni precipitated as Ni(OH)_2_, increasing the bpy/Ni ratio of the remaining solution and favoring the formation of stable Ni(bpy)_3_^2+^.^14^ At pH 12, the dissolved bpy/Ni ratio approached 3. Association of Ni(bpy)_3_^2+^ with ClO_4_^−^ forms the hydrophobic ion pair NIP,^22^ facilitating its extraction into DCE.

**Fig. 2:**
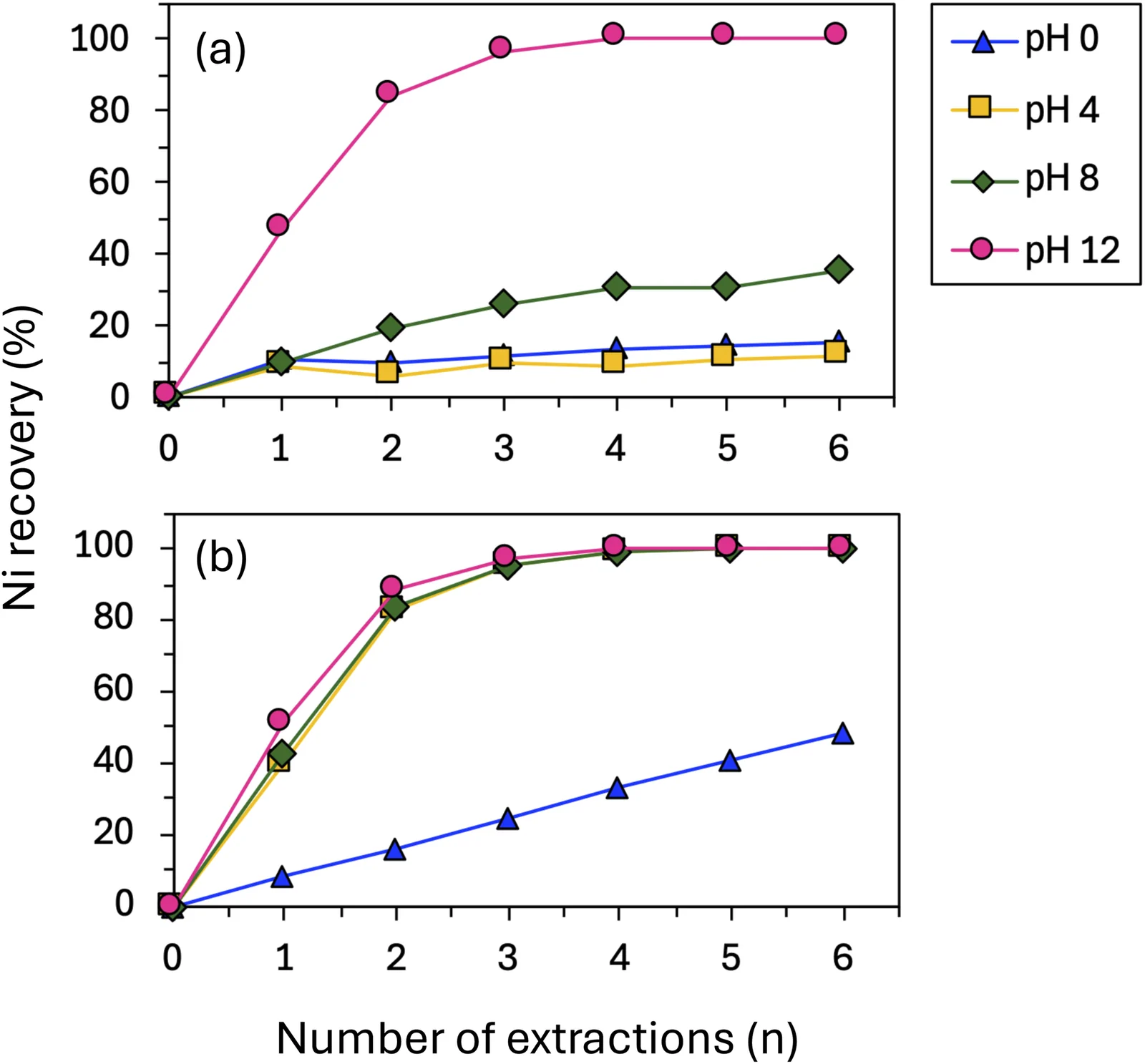
Effect of pH on the Ni recovery (%) for (a) SpC-1 and (b) SpC-3 by IPE.

SpC-3, with an initial bpy/Ni molar ratio of 3, exhibited a different pH dependence. At pH 0, only approximately 8% of Ni was recovered in the first extraction, and the cumulative recovery reached 48% after six extractions. In contrast, >99% recovery was achieved after five extractions at pH ≥ 4. Bipyridine undergoes successive protonation with reported p*K*_a_ values of approximately −0.52 and 4.35,^23^ and protonated bpy species become increasingly abundant under acidic conditions (SI Fig. S1). Protonation of the pyridyl N atoms competes with Ni^2+^ coordination and suppresses the formation of highly coordinated Ni–bpy complexes, particularly under strongly acidic conditions.^24^ Increasing pH therefore favors Ni–bpy complexation and subsequent extraction of NIP into DCE. Alkaline pretreatment substantially enhanced Ni recovery by IPE, particularly when the initial bpy/Ni ratio was below 3. After adjustment to pH 12, the dissolved Ni concentrations in SpC-1 and SpC-3 were approximately 3.8 and 38 mM, respectively. Despite this tenfold difference, both solutions exhibited similar extraction profiles and achieved >99% recovery after five sequential extractions, indicating that Ni–bpy speciation, rather than dissolved Ni concentration, was the primary factor controlling IPE within the concentration range examined.

Fig. 3 shows the effect of the added ClO_4_^−^/initial Ni molar ratio on Ni recovery from SpC-3 and SpC-A by IPE with five sequential extractions. Recovery increased sharply with increasing ClO_4_^−^ addition in both solutions, reaching approximately 80–90% at ClO_4_^−^/Ni ratios of 5–7 and >95% at higher ratios. Despite their different solution compositions, SpC-3 and SpC-A exhibited similar recovery trends. Thus, once conditions favored Ni(bpy)_3_^2+^ formation, the ClO_4_^−^/Ni ratio was a major factor controlling Ni recovery by IPE.

**Fig. 3:**
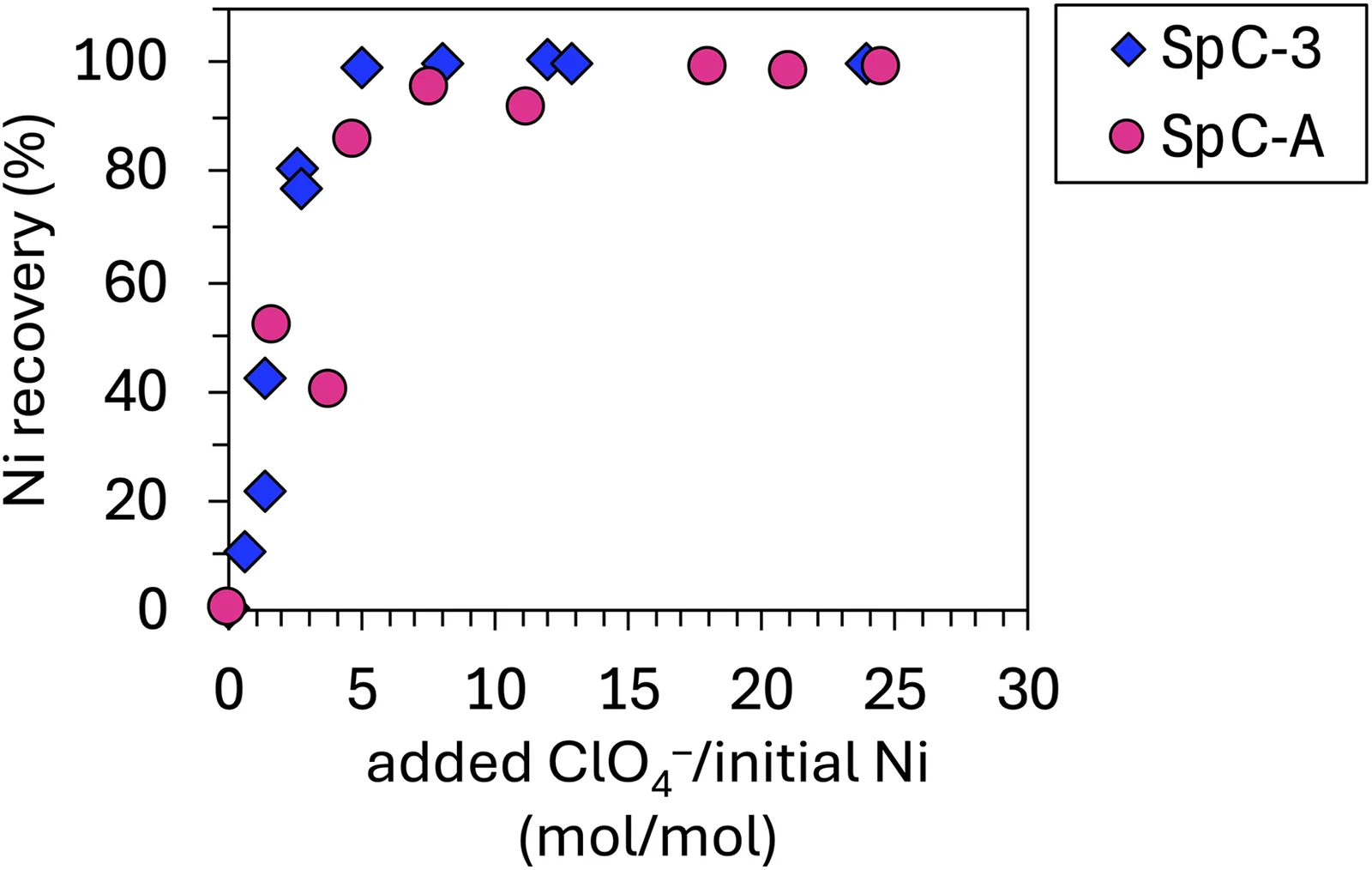
Ni recovery from SpC-3 and SpC-A by IPE as a function of the added ClO_4_^−^/initial Ni molar ratio. Five consecutive extractions were performed for each condition.

### 3.2. Recovery of Ni-bpy by IPP and chemical modeling

Fig. 4 shows Ni speciation (%) by the IPP method for SpC-A as a function of the ClO_4_^−^/Ni molar ratio. After alkaline pretreatment, the initial Ni concentration was approximately 10.6 mM, and NIP precipitation became apparent only at ClO_4_^−^/Ni ratios above approximately 20. A ratio of >50 was required to achieve >80% Ni recovery. Relatively large variations among replicate experiments were observed in the intermediate precipitation region (ClO_4_^−^/Ni ratios of approximately 20–50), where NIP precipitation increased rapidly with increasing ClO_4_^−^ addition. In contrast, the variation became small after the recovery reached a plateau. This behavior suggests that the observed variability was associated with the sensitivity of nucleation and crystal growth near the precipitation threshold. Larger excesses of ClO_4_^−^ were required for precipitation from SpC-A compared with the results of the IPE method, indicating that NIP precipitation is governed not only by the amount of added ClO_4_^−^ but also by the initial Ni concentration and the solution composition. To quantitatively interpret these effects, the precipitation equilibrium was further analyzed using PHREEQC.

**Fig. 4:**
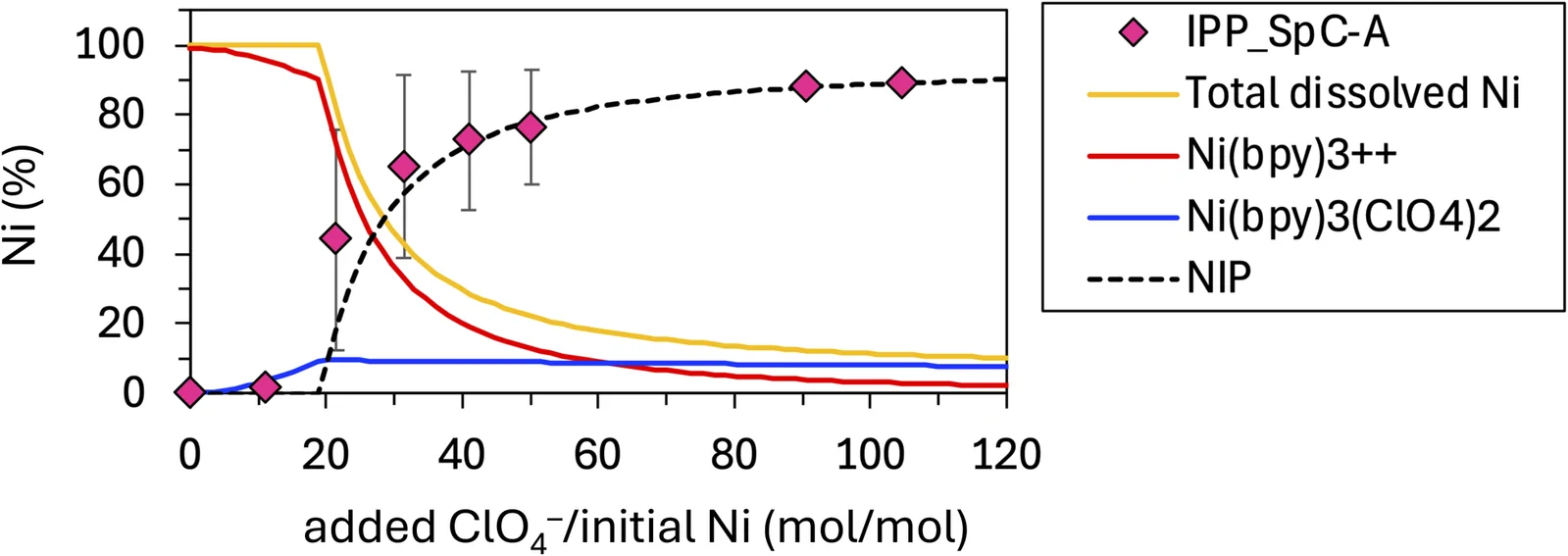
Experimental Ni recovery by IPP from SpC-A and Ni speciation simulated using PHREEQC as a function of the added ClO_4_^−^/initial Ni molar ratio. Plots and error bars represent the mean experimental recovery (*n* = 3) and standard deviation, respectively. Solid lines represent simulated dissolved Ni species, and the dashed line represents precipitated NIP.

The apparent solubility product of NIP (log *K*_sp,app_) was experimentally determined for different solution conditions (Table 2) and incorporated into the PHREEQC model. NIP exhibited very low solubility in pure water, corresponding to a log *K*_sp,app_ of −15.84. Its apparent solubility increased markedly under the alkaline conditions used for IPE and IPP, with log *K*_sp,app_ increasing to −8.87 at pH 12. The presence of MeOH further increased NIP solubility; increasing the MeOH content from 10 to 50 vol% resulted in log *K*_sp,app_ values ranging from −6.79 to −5.00, indicating that even relatively small amounts of MeOH substantially suppress NIP precipitation. The influence of NaCl was also examined because NaCl accumulates during alkaline neutralization of the strongly acidic catalyst solution. Under NaCl-saturated conditions, log *K*_sp,app_ increased further to −4.58. The high Cl^−^ concentration competes with the ion pairing of Ni(bpy)_3_^2+^ and ClO_4_^−^, thereby shifting the equilibrium toward Ni(bpy)_3_Cl_2_, which is a highly soluble species. In addition, the protonation and solution thermodynamics of bpy are known to depend on ionic strength and supporting electrolyte composition.^23,25^ The observed increase in log *K*_sp,app_ thus likely reflects changes in the overall ion pairing and solvation environment of Ni(bpy)_3_^2+^ and ClO_4_^−^ under highly concentrated NaCl conditions. SpC-A also contains organic components, including DMAc and 1,5-cyclooctadiene (COD), which may further influence NIP formation and precipitation. The effect of COD was initially examined; however, its low miscibility with the aqueous medium prevented preparation of a homogeneous model solution representative of SpC-A. The effect of residual organic components was therefore evaluated using DMAc, which is completely miscible with the aqueous phase. At a DMAc concentration of 900 mM, log *K*_sp,app_ increased by approximately one order of magnitude to −3.42, demonstrating that DMAc substantially increased the apparent solubility of NIP.

**Table 2:** The log *K*_sp,app_ values for the ion-pair precipitation corresponding to each solvent composition

	pH^a^	MeOH (v%)	NaCl^b^ (M)	DMAc (mM)	log *K*_sp,app_
Pure water	6 ± 1	—	—	—	−15.84
	12	—	—	—	−8.87
	12	10	—	—	−6.79
	12	20	—	—	−6.39
	12	50	—	—	−5.00
NaOH (*ca.* 0.01 M)	12	50	0.02	—	−4.94
	12	50	0.2	—	−4.61
	12	50	2	—	−4.58
	12	50	2	90	−4.43
	12	50	2	300	−4.03
	12	50	2	900	−3.42

a The pH values were adjusted with NaOH.

b The saturated NaCl concentration in 50 vol% MeOH was approximately 2.0 M, consistent with reported solubility data for NaCl in water–methanol mixtures.^26^

The model incorporating this experimentally determined log *K*_sp,app_ successfully reproduced the onset of NIP precipitation but overestimated Ni recovery at high ClO_4_^−^/Ni ratios (SI Fig. S2 and Text S1). However, Ni recovery approached a plateau of approximately 90% even at ClO_4_^−^/Ni ratios above 80, suggesting that a fraction of the ion-paired complex remained dissolved even under conditions thermodynamically favorable for precipitation. A dissolved ion-pair species, Ni(bpy)_3_(ClO_4_)_2_(aq), was thus introduced into the PHREEQC model:Ni(bpy)_3_^2+^ + 2ClO_4_^−^ ⇄ Ni(bpy)_3_(ClO_4_)_2_ (aq).

The corresponding formation constant, *K*_IP_, was defined as follows:
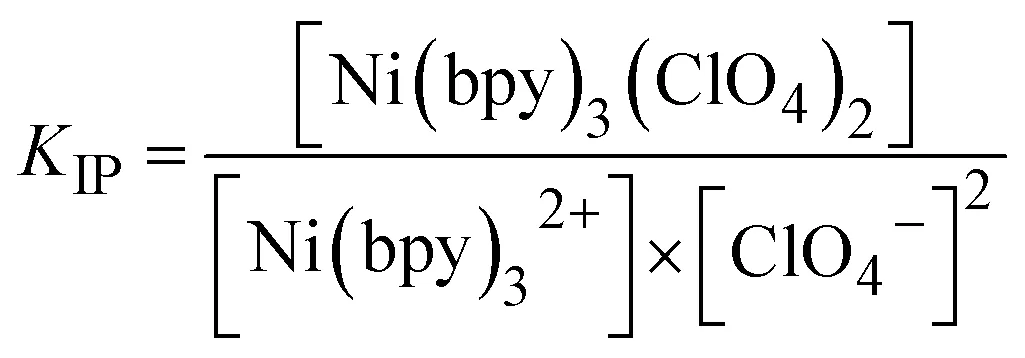


Because the dissolved ion-pair species could not be independently quantified, the value of *K*_IP_ was treated as an adjustable model parameter. Simulations were performed over a range of log *K*_IP_ values and compared with the experimental precipitation behavior of SpC-A (SI Fig. S2). The experimental data were reproduced when log *K*_IP_ was in the range of approximately 0.42–1.42, and a value of 0.62 provided the best fit based on least-squares minimization. Incorporation of this dissolved ion-pair species enabled the model to reproduce not only the onset of precipitation but also the recovery plateaus observed for SpC-A. Although deviations remained in the intermediate precipitation region, where nucleation and crystal-growth kinetics likely affected the approach to equilibrium, the model adequately reproduced the thermodynamically relevant precipitation threshold and attainable recovery limit.

Fig. 5 compares the experimental Ni recovery by IPP from SpC-3 with the PHREEQC simulations at different initial Ni concentrations. The ClO_4_^−^/Ni ratio required for NIP precipitation strongly depended on the initial Ni concentration. At an initial Ni concentration of 38.3 mM, approximately 96% Ni recovery was achieved at a ClO_4_^−^/Ni ratio of 6 (Fig. 5(a)). As the initial Ni concentration decreased, substantially larger excesses of ClO_4_^−^ were required. Approximately 12 and 30 ClO_4_^−^/Ni ratios were required to complete the recovery for initial Ni concentrations of 19.4 and 10.7 mM, respectively (Fig. 5(b) and (c)).

**Fig. 5:**
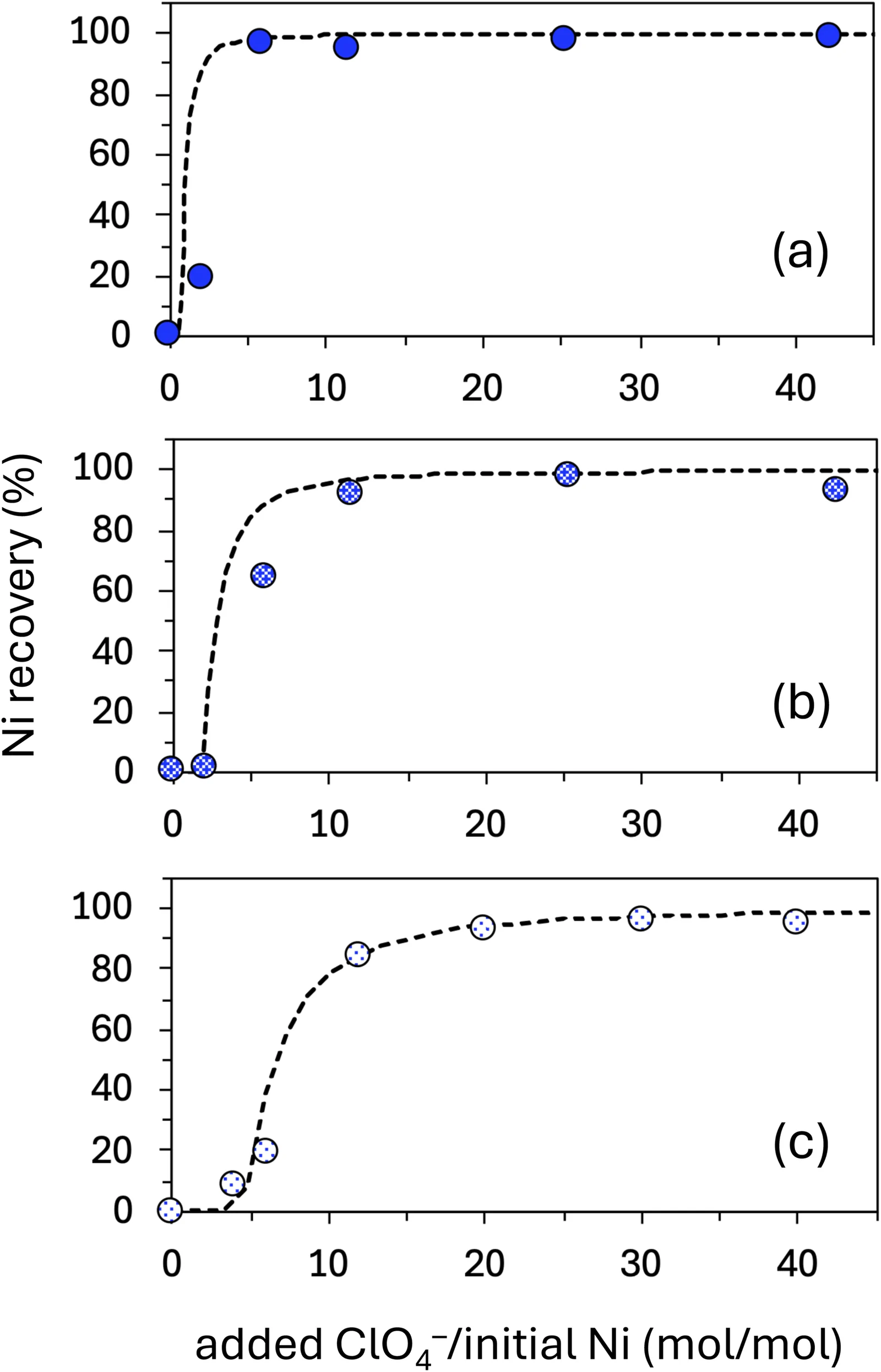
Experimental (plots) and simulated (dashed lines) Ni recovery by IPP from SpC-3 as a function of the added ClO_4_^−^/initial Ni molar ratio at initial Ni concentrations of (a) 38.3 mM, (b) 19.4 mM, and (c) 10.7 mM.

This concentration dependence can be explained by the precipitation equilibrium of NIP. At a given ClO_4_^−^/Ni ratio, decreasing the initial Ni concentration decreases the absolute concentrations of Ni(bpy)_3_^2+^, making the ion activity product less likely to reach *K*_sp,app_. Consequently, a larger excess of ClO_4_^−^ is required to induce NIP precipitation at lower Ni concentrations. The PHREEQC model developed using the equilibrium parameters (log *K*_sp,app_ = −4.58 from Table 2, and log *K*_IP_ = 0.62) successfully reproduced the experimental recovery profiles at all three initial Ni concentrations, demonstrating that the observed concentration dependence arises from the precipitation and ion-pair equilibria rather than from changes in the fitted equilibrium parameters.

Ni recovery by IPP was governed by the competition between precipitation of solid NIP, represented by *K*_sp,app_, and stabilization of the dissolved Ni–bpy–ClO_4_ ion pair, represented by *K*_IP_. In contrast, during IPE, NIP formed in the aqueous phase is continuously transferred to the DCE phase, shifting the ion-pair equilibrium toward further NIP formation. Consequently, IPE achieved >99% Ni recovery at substantially lower ClO_4_^−^/Ni ratios and was less sensitive to the initial Ni concentration than IPP under the conditions examined.

### 3.3. Characterization of NIP and counterion conversion

Photographs of NIP recovered from SpC-3 and SpC-A by IPE and IPP, together with the product after ClO_4_^−^-to-Cl^−^ counterion exchange and independently synthesized Ni(bpy)_3_Cl_2_, are shown in SI Fig. S3. The recovered NIP exhibited a reddish-orange appearance, whereas the product after counterion exchange was pink-purple, similar in appearance to the independently synthesized Ni(bpy)_3_Cl_2_ reference. The NIP recovered by IPE and IPP was characterized by ATR-FTIR spectroscopy (Fig. 6). Free bpy exhibited characteristic pyridine-ring skeletal vibration bands at 1578, 1556, 1450, and 1415 cm^−1^ (Fig. 6(a)), consistent with previously reported vibrational assignments for bpy.^27,28^ Upon coordination with Ni^2+^, these bands shifted to approximately 1600, 1578, 1466, and 1445 cm^−1^, respectively, as observed for the Ni(bpy)_3_Cl_2_ reference. These spectral changes provide a characteristic fingerprint for distinguishing coordinated bpy from free bpy.^14,28,29^ The products recovered from both SpC-3 and SpC-A by IPE and IPP exhibited spectral patterns similar to that of the Ni(bpy)_3_Cl_2_ reference in the pyridine-ring vibration region (Fig. 6(b)). This similarity indicates that bpy remained coordinated to Ni during both recovery processes. In addition, the recovered products exhibited an intense absorption band in the approximately 1000–1150 cm^−1^ region that was absent from the Ni(bpy)_3_Cl_2_ reference (Fig. 6(c)(d)). This band is characteristic of the asymmetric stretching vibration of ClO_4_^−^,^30,31^ indicating that ClO_4_^−^ was associated with the Ni–bpy complex. These ATR-FTIR results support the assignment of the recovered products to the perchlorate-containing Ni–bpy ion pair, Ni(bpy)_3_(ClO_4_)_2_.

**Fig. 6:**
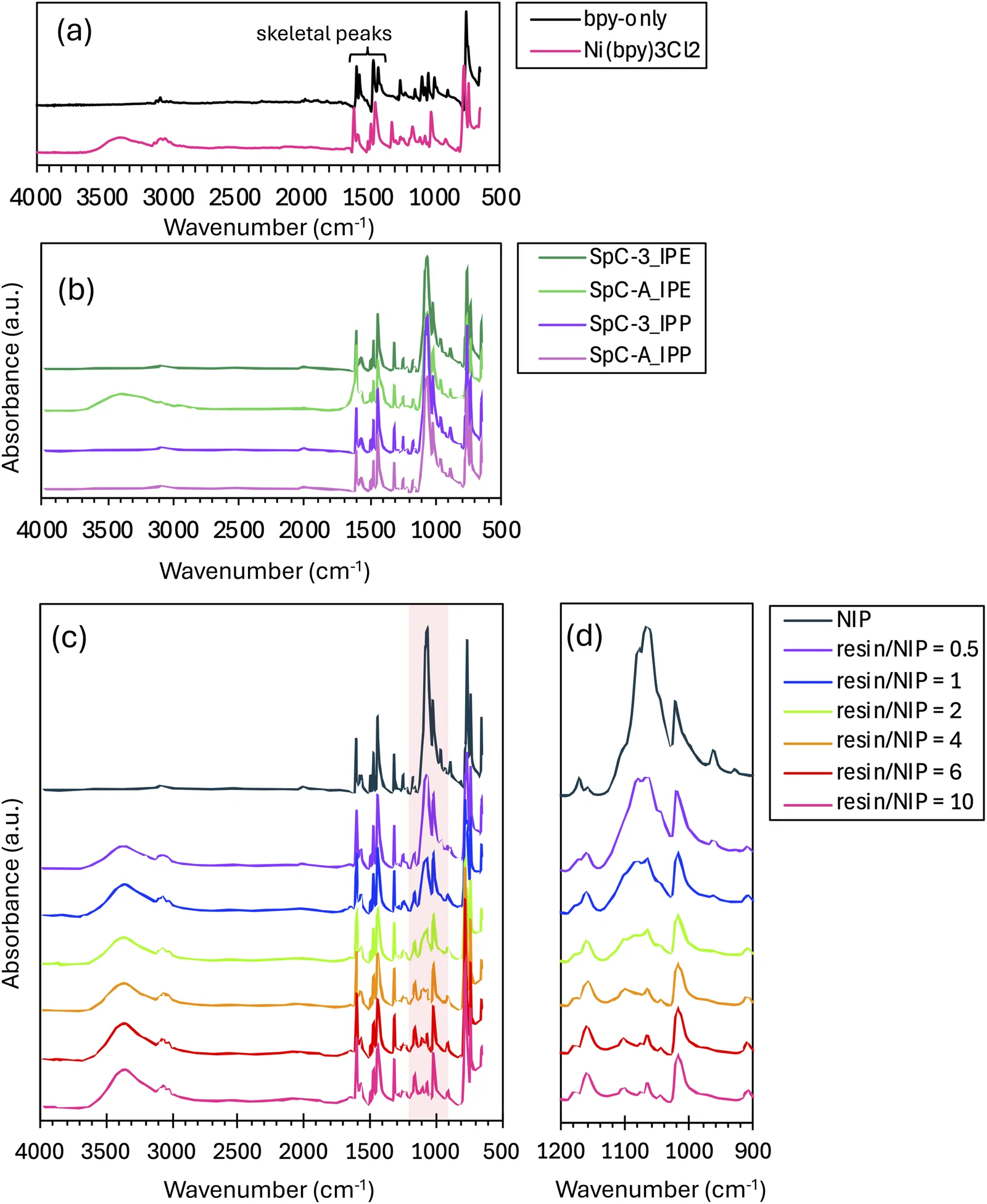
ATR-FTIR spectra of (a) free bpy and the Ni(bpy)_3_Cl_2_ reference, (b) Ni–bpy complexes recovered from SpC-3 and SpC-A by IPE and IPP, and (c) NIP before and after counterion exchange using an anion-exchange resin at resin/NIP mass ratios of 0.5–10. (d) Expanded spectra of the ClO_4_^−^ vibration region (900–1200 cm^−1^) shown in (c).

Although NIP can be efficiently recovered by IPE or IPP, its low water solubility limits its direct reuse as a homogeneous Ni–bpy catalyst. Therefore, counterion exchange from ClO_4_^−^-to Cl^−^ was performed using a Cl^−^-form anion-exchange resin. Fig. 7 shows the ClO_4_^−^-to-Cl^−^ exchange efficiency as a function of the resin/NIP ratio. The apparent exchange efficiency increased from 72.1% at a resin/NIP ratio of 1 to 88.4% at a ratio of 2 and reached 98.1% at a ratio of 4. Further increases in the resin dosage resulted in little change, with apparent exchange efficiencies of 97.1–98.8% at resin/NIP ratios of 6–10. Background experiments were performed by the same procedure using independently synthesized Ni(bpy)_3_Cl_2_. The result revealed that approximately 1.9 ± 0.9% (*n* = 3) of Ni was transferred to the DCE phase even in the absence of ClO_4_^−^, suggesting that the residual 1–3% apparent non-exchanged fraction observed at resin/NIP ratios of ≥4 is within the uncertainty of the analytical method. Thus, counterion exchange can be regarded as essentially quantitative at a resin/NIP mass ratio of ≥4 under the conditions examined. The counterion conversion was further evaluated by ATR-FTIR spectroscopy (Fig. 6(c) and (d)). The intensity of the ClO_4_^−^-related band at 1000–1150 cm^−1^ progressively decreased with increasing resin/NIP ratio and became negligible at higher resin dosages. In contrast, the characteristic skeletal vibration bands of coordinated bpy were retained throughout the ion-exchange treatment. These spectral changes indicate the removal of ClO_4_^−^ while preserving the coordinated state of bpy in the recovered Ni complex. The disappearance of the ClO_4_^−^ band was consistent with the >95% exchange efficiency independently estimated from the extraction analysis.

**Fig. 7:**
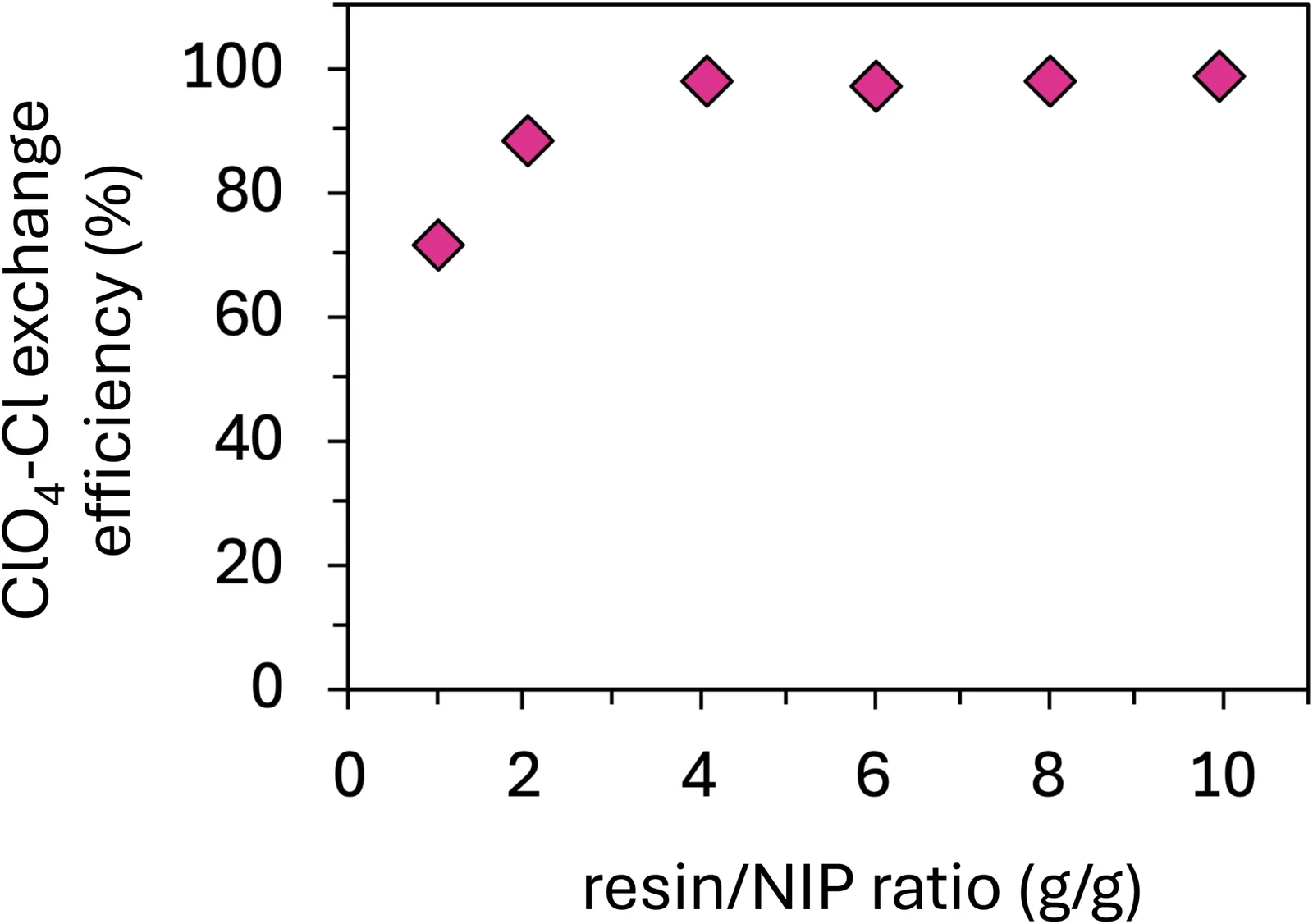
Effect of the resin/NIP ratio (g g^−1^) on the ClO_4_^−^ to Cl^−^ exchange efficiency by anion-exchange treatment of NIP.

These results demonstrate that the counterion-exchange step converts poorly water-soluble NIP into a water-soluble form without appreciable loss of the characteristic coordination features of the Ni–bpy complex, thereby providing a process for recovering the catalyst complex in a form suitable for reuse.

## 4. Conclusion

This study developed ion-pair methods for recovering Ni–bpy complexes from spent catalyst solutions while retaining bpy in its coordinated form. Alkaline pretreatment promoted the formation of highly coordinated Ni–bpy species by removing free or weakly complexed Ni as Ni(OH)_2_, thereby substantially improving subsequent ion-pair formation with ClO_4_^−^. The resulting perchlorate-containing Ni–bpy ion pair, Ni(bpy)_3_(ClO_4_)_2_ (NIP), could be recovered either by liquid–liquid extraction with DCE (IPE) or by direct precipitation (IPP). High Ni recovery (>99%) was achieved by IPE at ClO_4_^−^/Ni ratios greater than 10 across the Ni concentrations and solution compositions examined. In contrast, IPP exhibited a strong dependence on the initial Ni concentration and solution composition. Experimentally determined apparent solubility products, log *K*_sp,app_, showed that NIP solubility increased substantially in the presence of MeOH, NaCl, and DMAc. Furthermore, incorporating a dissolved Ni(bpy)_3_(ClO_4_)_2_ ion-pair species into the PHREEQC model successfully reproduced both the onset of NIP precipitation and the experimentally observed recovery plateaus. These results indicate that competition between dissolved and precipitated ion pairs limits Ni recovery by IPP, whereas continuous transfer of NIP into the organic phase enables IPE to overcome this limitation. ATR-FTIR analysis confirmed that the recovered products retained the characteristic spectral features of coordinated bpy and contained ClO_4_^−^, supporting their assignment as NIP. Furthermore, more than 95% of the ClO_4_^−^ counterions could be exchanged with Cl^−^ using a Cl^−^-form anion-exchange resin, while the characteristic spectral features of coordinated bpy were retained. This counterion conversion transformed poorly water-soluble NIP into a water-soluble form suitable for subsequent reuse. The proposed process provides an effective strategy for recovering Ni together with its functional ligand from spent catalyst solutions, and offers a potential route toward closed-loop recycling of homogeneous metal–ligand catalysts.

## Author contributions

H. Iwai: conceptualization, methodology, investigation, formal analysis, writing – original draft. Y. Takaya: conceptualization, writing – review & editing. N. Yokota: investigation, writing – review & editing. Y. Takahashi: investigation, writing – review & editing. C. Tokoro: supervision, conceptualization, writing – review & editing. All authors reviewed and approved the final manuscript.

## Conflicts of interest

The authors declare that they have no known competing financial interests or personal relationships that could have influenced the work reported in this study.

## Supplementary Material

RA-016-D6RA08431A-s001

## Data Availability

The data supporting this article have been included as part of the supplementary information (SI). Supplementary information: supplementary data to this article can be found online. See DOI: https://doi.org/10.1039/d6ra08431a.
