# Supplementary material for: Ion-pair-assisted recovery of nickel(ii) tris(2,2′-bipyridine) complexes from spent catalysts by extraction and precipitation
Source: RSC Adv. 2026 Oct 22. Online ahead of print. doi: 10.1039/d6ra08431a (PMC13687800; doi:10.1039/d6ra08431a)
Supplement: Supplementary file 1 [file RA-OLF-D6RA08431A-s001.pdf]

Supplementary Information (SI) for RSC Advances.
This journal is © The Royal Society of Chemistry

Supporting Information

Ion-pair-assisted recovery of nickel(II) tris(2,2′-bipyridine) complexes from spent catalysts by extraction and precipitation

Hisanori Iwai[a], Yutaro Takaya[b], Naoki Yokota[c], Yuko Takahashi[c], Chiharu Tokoro[a,b*]

[a] Department of Resources and Environmental Engineering, Faculty of Science and Engineering, Waseda University, 169-8555 Tokyo, Japan
[b] Department of System Innovation, Faculty of Engineering, The University of Tokyo, 113-8656 Tokyo, Japan
[c] Next-Generation Technology Design and Creation, TAKAHATA PRECISION Co., Ltd., 160-0023 Tokyo, Japan

*Corresponding author: tokoro@waseda.jp

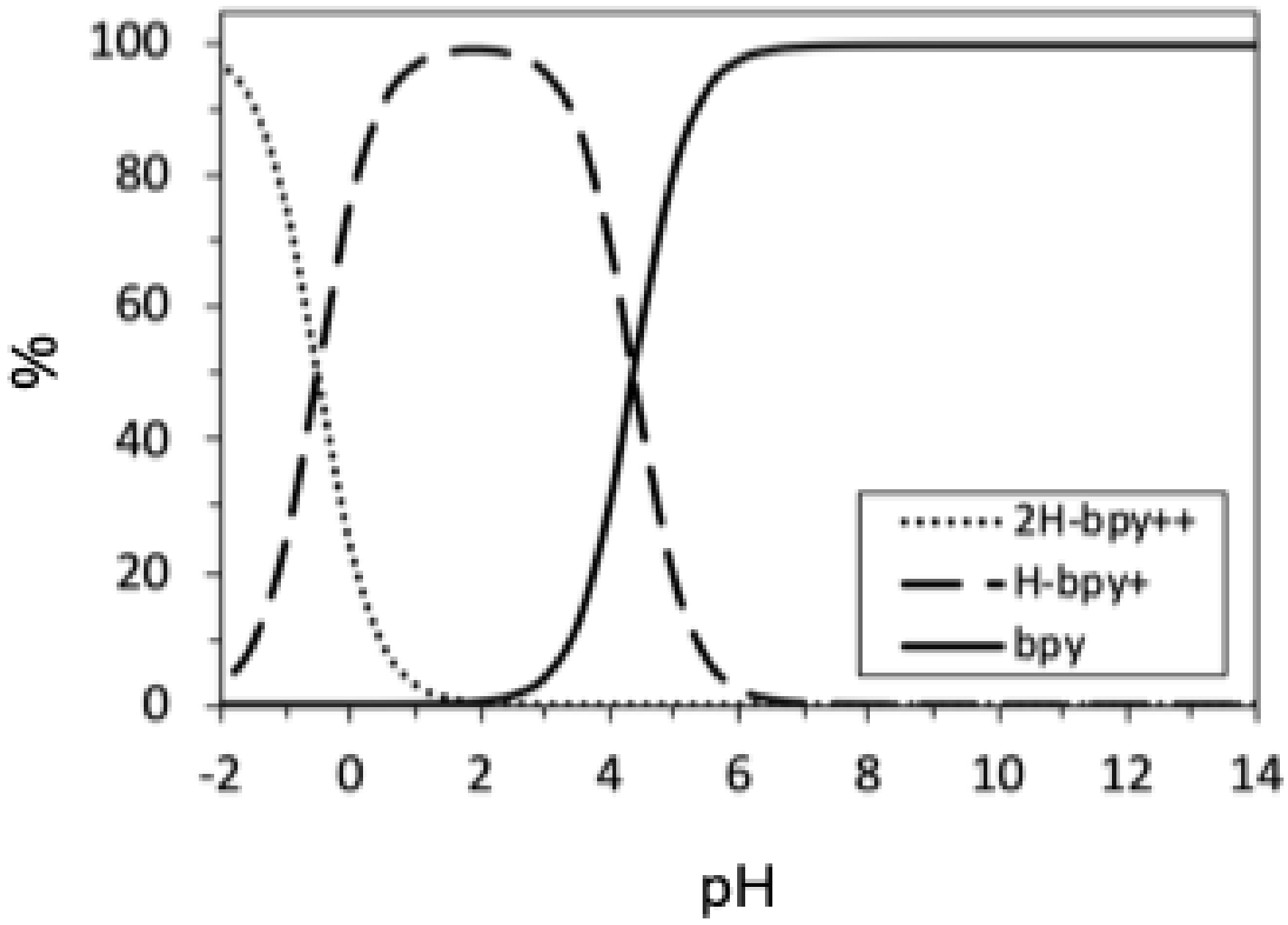

Figure S1. Calculated pH-dependent distribution of protonation states of 2,2′-bipyridine (bpy) based on reported acid dissociation constants.[23]

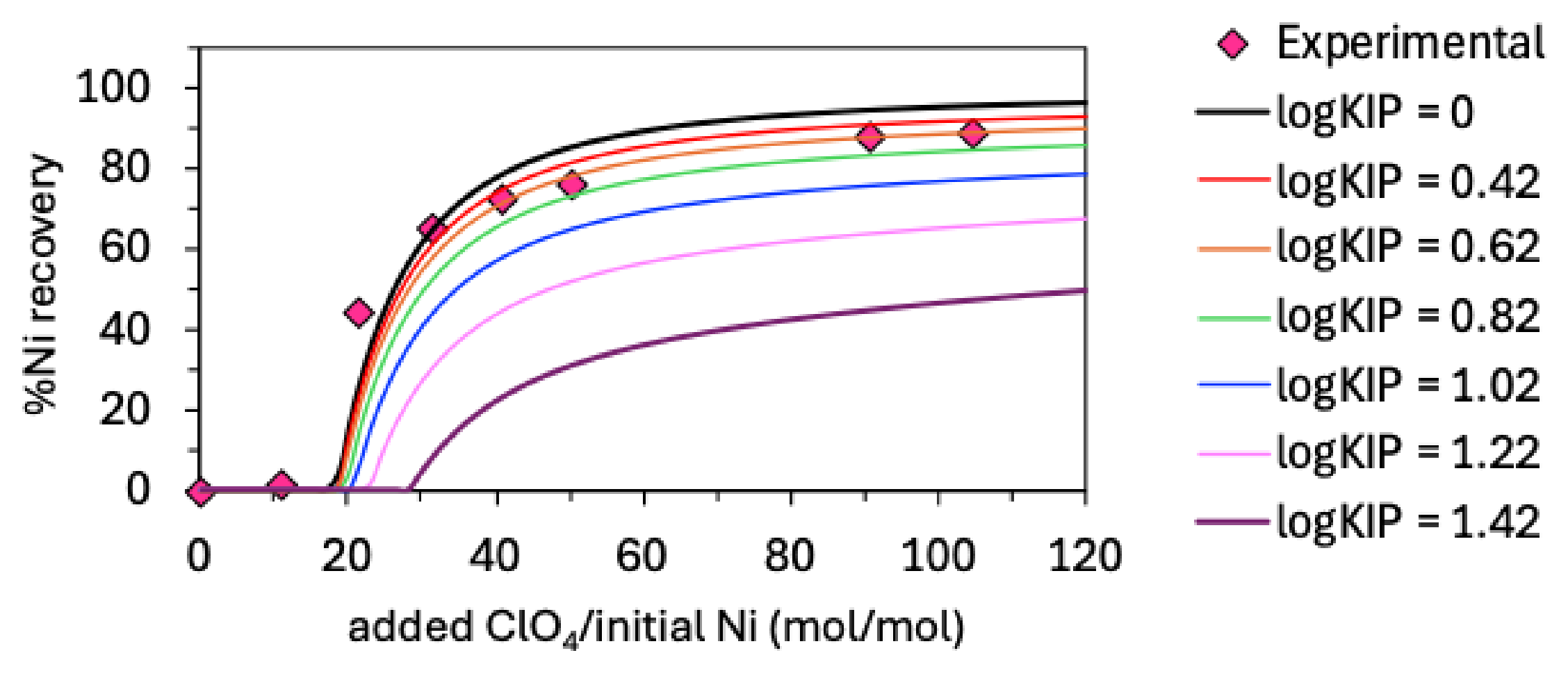

Figure S2. Effect of the formation constant of the dissolved ion pair, $Ni(bpy)_3(ClO_4)_2(aq)$, on the simulated Ni recovery by ion-pair precipitation (IPP) for SpC-A. Solid lines represent PHREEQC simulations obtained using different logK_{IP} values, and symbols represent the experimental data.

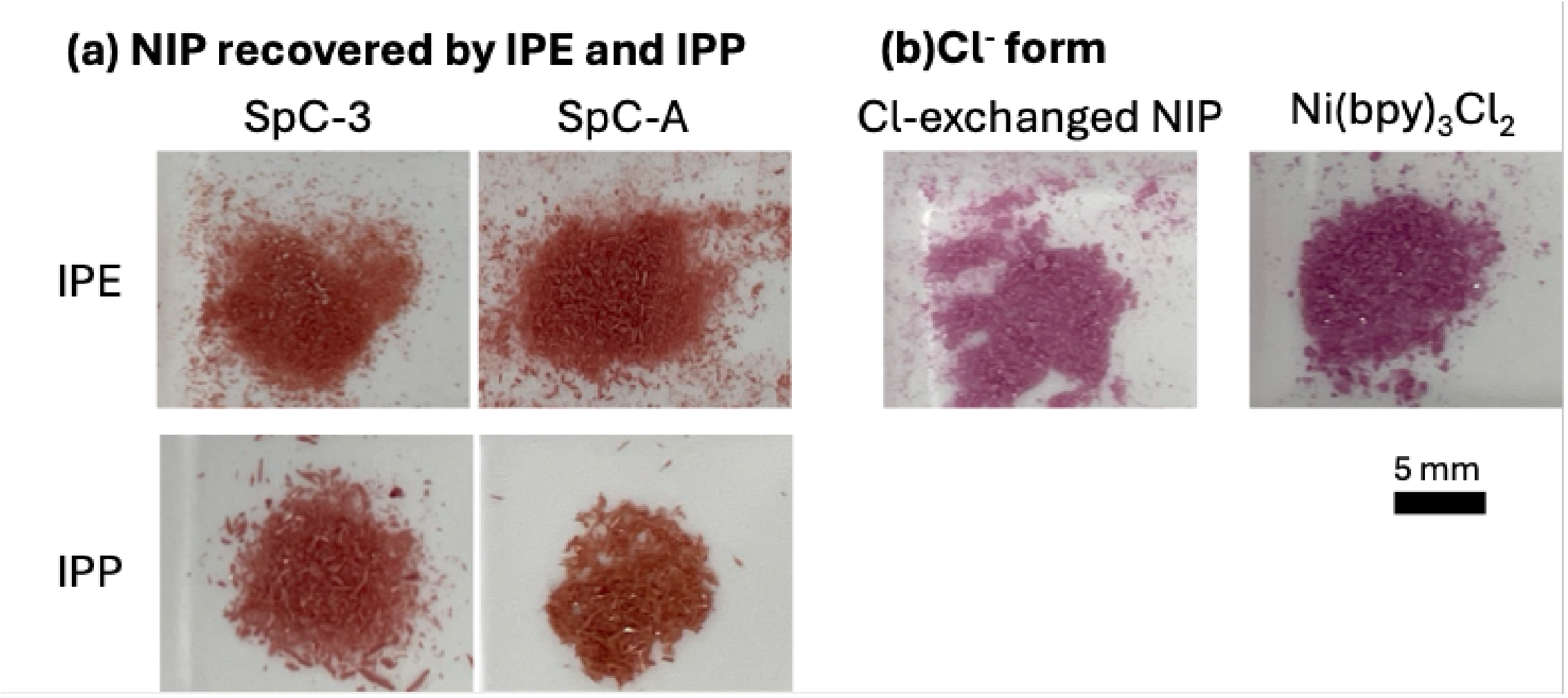

Figure S3. Photographs of (a) NIP recovered from SpC-3 and SpC-A by IPE and IPP and (b) NIP after ClO_4^--to-Cl^- counterion exchange and independently synthesized $Ni(bpy)_3Cl_2$ used as a reference.

Text S1. PHREEQC code for simulating the IPP performance.

```
##############################
###    PHREEQC code for IPP    ###
##############################
SOLUTION_MASTER_SPECIES
        Bpy            Bpy            0        Bpy            156.18
        A              A-             0        A-             99
#2,2'-bipyridine and perchlorate ion are defined as Bpy and A.

SOLUTION_SPECIES
Bpy = Bpy
        log_k       0
Bpy + Ni+2 = Ni(Bpy)+2
        log_k       6.92
Bpy + Ni(Bpy)+2 = Ni(Bpy)2+2
        log_k       6.77
Bpy + Ni(Bpy)2+2 = Ni(Bpy)3+2
        log_k       5.89
A- = A-
        log_k       0
2A- + Ni(Bpy)3+2 = Ni(Bpy)3(A)2
        log_k       0.62

PHASES
NIP
        Ni(Bpy)3(A)2 = Ni(Bpy)3+2 + 2A-
        log_k       -3.42
#Select the suitable value for the medium composition from Table 2.

EQUILIBRIUM_PHASES 1
        NIP      0 0

SOLUTION 1
        temp        25
        pH          12
```

```
    pe           4
    redox        pe
    units        mmol/kgw
    density      1
    Bpy          34.5
    Cl(-1)       2000
    A        0
    Na           2000
    Ni           11.5
    -water       1 # kg
# Define the concentration of Ni and bpy.

REACTION 1
    A            1
1800 millimoles in 100 steps
# Define the total amount and the step of perchlorate ion that will be added.

USER_GRAPH 1
    -headings                  A Ni++ Ni(Bpy)3+2 Ni(Bpy)3(A)2 Ni(OH)2 NIP
    -axis_titles               "A" "Ni" "Precipitate"
    -chart_title               "Ni-IPP"
    -initial_solutions         true
    -connect_simulations       true
    -plot_concentration_vs     x
  -start
10 GRAPH_X TOT("A")
20 GRAPH_Y TOT("Ni") MOL("Ni(Bpy)3+2") MOL("Ni(Bpy)3(A)2")
30 GRAPH_SY EQUI("Ni(OH)2") EQUI("NIP")
  -end
    -active                    true
```
